# Adult ciliary epithelial stem cells generate functional neurons and differentiate into both early and late born retinal neurons under non-cell autonomous influences

**DOI:** 10.1186/1471-2202-14-130

**Published:** 2013-10-22

**Authors:** Carolina Beltrame Del Debbio, Xu Peng, Huangui Xiong, Iqbal Ahmad

**Affiliations:** 1 Department of Ophthalmology and Visual Sciences, University of Nebraska Medical Center, Durham Research Center 1, Room 4044, 985840 Nebraska Medical Center, Omaha, NE 68198-5840, USA; 2 Department of Pharmacology and Experimental Neuroscience, University of Nebraska Medical Center, Omaha, Nebraska 68198-5840, USA

**Keywords:** Stem cells, Ciliary epithelium, Photoreceptors, Retinal ganglion cells, Cell therapy, Retina

## Abstract

**Background:**

The neural stem cells discovered in the adult ciliary epithelium (CE) in higher vertebrates have emerged as an accessible source of retinal progenitors; these cells can self-renew and possess retinal potential. However, recent studies have cast doubt as to whether these cells could generate functional neurons and differentiate along the retinal lineage. Here, we have systematically examined the pan neural and retinal potential of CE stem cells.

**Results:**

Molecular and cellular analysis was carried out to examine the plasticity of CE stem cells, obtained from mice expressing green fluorescent protein (GFP) under the influence of the promoter of the rod photoreceptor-specific gene, *Nrl*, using the neurospheres assay. Differentiation was induced by specific culture conditions and evaluated by both transcripts and protein levels of lineage-specific regulators and markers. Temporal pattern of their levels were examined to determine the expression of genes and proteins underlying the regulatory hierarchy of cells specific differentiation *in vitro*. Functional attributes of differentiation were examined by the presence of current profiles and pharmacological mobilization of intracellular calcium using whole cell recordings and Fura-based calcium imaging, respectively. We demonstrate that stem cells in adult CE not only have the capacity to generate functional neurons, acquiring the expression of sodium and potassium channels, but also respond to specific cues in culture and preferentially differentiate along the lineages of retinal ganglion cells (RGCs) and rod photoreceptors, the early and late born retinal neurons, respectively. The retinal differentiation of CE stem cells was characterized by the temporal acquisition of the expression of the regulators of RGCs and rod photoreceptors, followed by the display of cell type-specific mature markers and mobilization of intracellular calcium.

**Conclusions:**

Our study demonstrates the bonafide retinal potential of adult CE stem cells and suggests that their plasticity could be harnessed for clinical purposes once barriers associated with any lineage conversion, i.e., low efficiency and fidelity is overcome through the identification of conducive culture conditions.

## Background

More than a decade ago two labs independently discovered that the adult rodent CE, a tissue of neuroectodermal origin between the retina and retinal pigment epithelium (RPE), contained a small subset of cells, which displayed neural stem cell properties *in vitro*[1,2]. These cells, when removed from their niche and cultured in the presence of mitogens proliferated and generated clonal neurospheres. Cells in adult CE neurospheres were proliferative, expressed pan-neural and retinal progenitor markers and differentiated along pan-neural and retinal lineages [1-5]. Further characterization revealed that these are a rare population of adult CE cells and unlike progenitors in the embryonic retina they displayed a cardinal feature of stem cells, i.e., they could self-renew [1,3]. The presence of such cells in rodent eyes was re-confirmed [6-11] and the evidence for their presence in postnatal chicken [12], rabbit [13], porcine [14,15], humans [9,16-18] and monkeys [18] emerged, suggesting evolutionary conservation of such cell population in adult vertebrate eyes. Further examination of their properties in rodents showed their relationship with retinal progenitor cells at the transcriptome level [8,19]. The conservation of adult CE stem cells in higher vertebrates, their retinal progenitor properties and ability to differentiate along retinal lineage *in vitro*[1,2,4,13,16,20] and *in vivo*[20,21] suggested that these cells may be analogous to regenerative cells in the ciliary margin zone of the lower vertebrates [22-24]. Thus, these relatively accessible adult stem cells were posited as an alternate heterologous source from which progenitors with retinal potential may be derived for retinal cell therapy [25]. However, two recent reports suggested that adult CE stem cells do not possess neural or retinal potential. Cicero et al., 2009 [26], based on the pigmented and epithelial features of these cells, questioned their characterization as retinal stem cells and reported that they lack the potential to differentiate along bonafide neural and retinal lineages. Gualdoni et al., 2010 [27], using *Nrl-GFP* mice [28], for genetic labeling of adult CE cells, enabling their lineage tracing along the rod photoreceptor lineage, and monolayer adherent culture concluded that these cells fail to differentiate into rod photoreceptors. Consequently, we have determined whether or not adult CE stem cells possess the capacity for pan-neuronal and retinal differentiation by systematically examining the temporal acquisition of the expression of cell-type specific regulators and phenotype specific markers along with the display of cell-type specific functional attributes. Our study not only confirms that these cells generate functional neurons but also demonstrates that like retinal progenitors they respond to specific culture conditions simulating the environment during retinal histogenesis and differentiate into both early and late born retinal neurons with functional attributes. Thus our study demonstrates that the adult CE stem cells do possess retinal potential and suggests that their plasticity could be harnessed for potential clinical purposes once the barriers associated with lineage conversion, i.e., low efficiency and fidelity, are overcome through the identification of conducive culture conditions.

## Results

**Figure 1:**
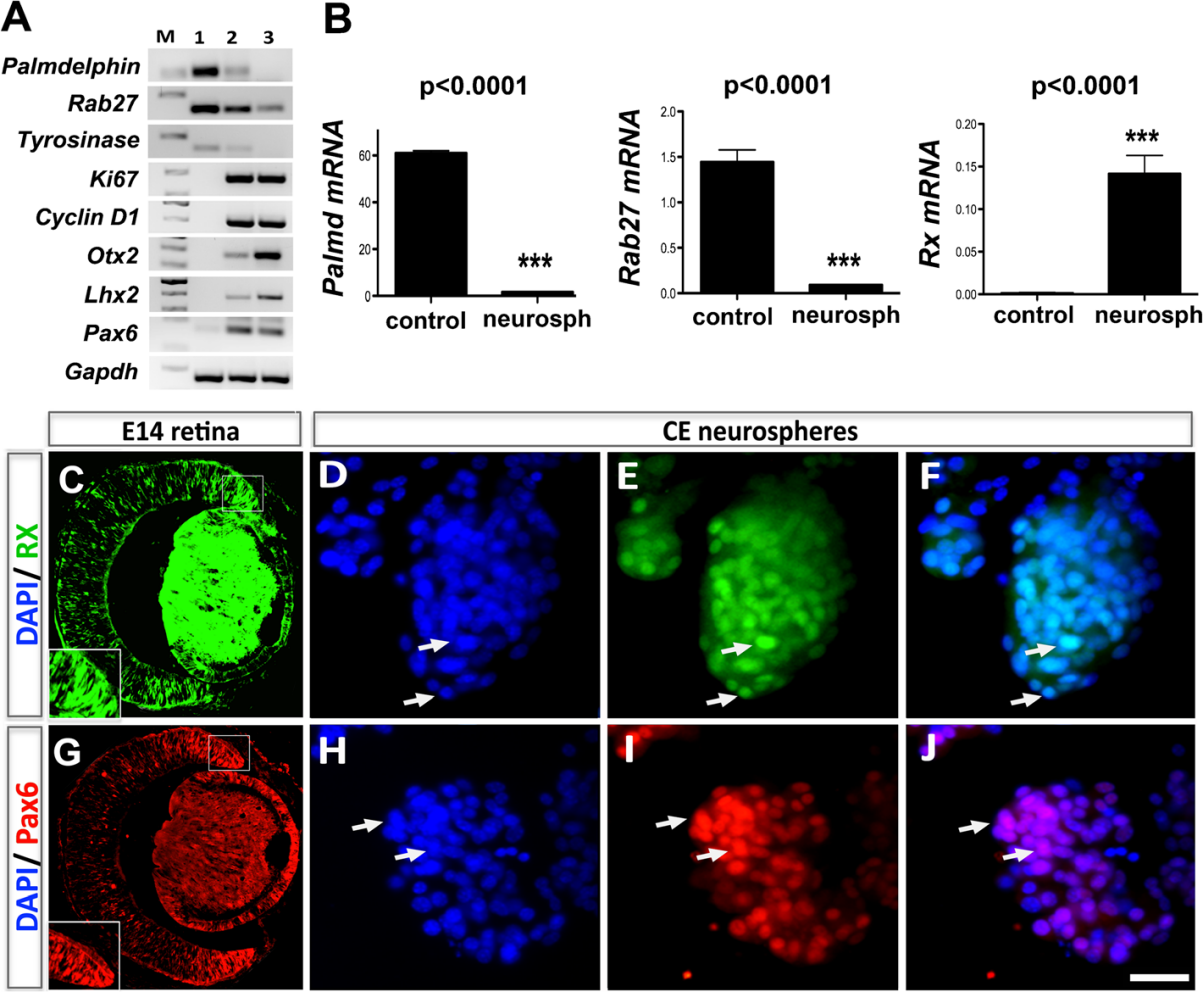
**Cells in CE neurospheres display retinal progenitor properties.** Adult CE cells were cultured in the presence of mitogens to generate neurospheres and the acquisition of retinal properties was examined. RT-PCR revealed that the levels of CE-specific transcripts (*Tyrosinase, Palmdelphin,* and *Rab27*) decreased in neurospheres with the time (lane 1 = untreated CE cells, lane 2 = 3 days old neurospheres, lane 3 = 6 days old neurospheres). In contrast, cell proliferation-(*Ki67 and Cyclin D1,)* and retinal progenitor (*Otx2, Lhx2 and Pax6*)-specific transcript levels increased temporally in neurospheres **(A)**. Q-PCR analysis corroborated the decrease in the levels of *Palmdelphin*, and *Rab27* transcripts and detected *Rx* transcripts in neurospheres **(B)**. Immunofluorescence analysis revealed that cells in CE neurospheres were immunoreactive for Rx **(D, E, F)** and Pax6 **(H, I, J)** as retinal progenitors in E14 retina **(C and G)**. Scale = 50 μm.

Experiments were carried out on CE cell dissociates, obtained from *Nrl-GFP* mice [28]. Since the fidelity of lineage and sub-lineage conversion depends upon re-programming of gene expression, we first examined the temporal expression patterns of select CE- and retinal progenitor-specific genes during the neurosphere assay by regular PCR (Figure 1A). We observed that cells in the beginning of the assay were characterized by CE-specific transcripts, *Palmdelphin*, *Rab27*, and *Tyrosinase*, and their levels decreased temporally in CE neurospheres. In contrast, transcripts corresponding to regulators of cell cycle, *Ki67* and *CyclinD1*, and of retinal progenitors *Otx2*, *Lhx2*, and *Pax6*, which were undetectable in CE cells, temporally increased in neurospheres (Figure 1A). The two amplification bands in Pax6 PCR results represent two transcripts corresponding to Pax6 isoforms [29]. A more sensitive analysis of transcripts by Q-PCR to determine the erasure of parental gene expression revealed that although levels of *Palmdelphin* (*Palmd*, 95%, p < 0.0001) and *Rab27* (90%, p < 0.0001) expression decreased precipitously, their residual expression remained in neurospheres on the 6^th^ day in culture (Figure 1B). Q-PCR revealed the expression of *Rx*, a critical regulator of retinal progenitors and which was undetectable by regular PCR, in CE neurospheres (Figure 1B). That the acquired expression of retinal progenitor-specific genes translated into respective proteins in adult CE neurospheres was revealed by immunoreactivities corresponding to Rx (Figure 1C-F) and Pax6 (Figure 1G-J). The proportion of cells expressing Rx and Pax6 immunoreactivities was 63 ± 0.65% and 56 ± 0.52%, respectively. Together, these observations suggested that cells that generated CE neurospheres displayed the properties of their resident epithelium, which were progressively attenuated as retinal progenitors markers were temporally expressed *in vitro*.

**Figure 2:**
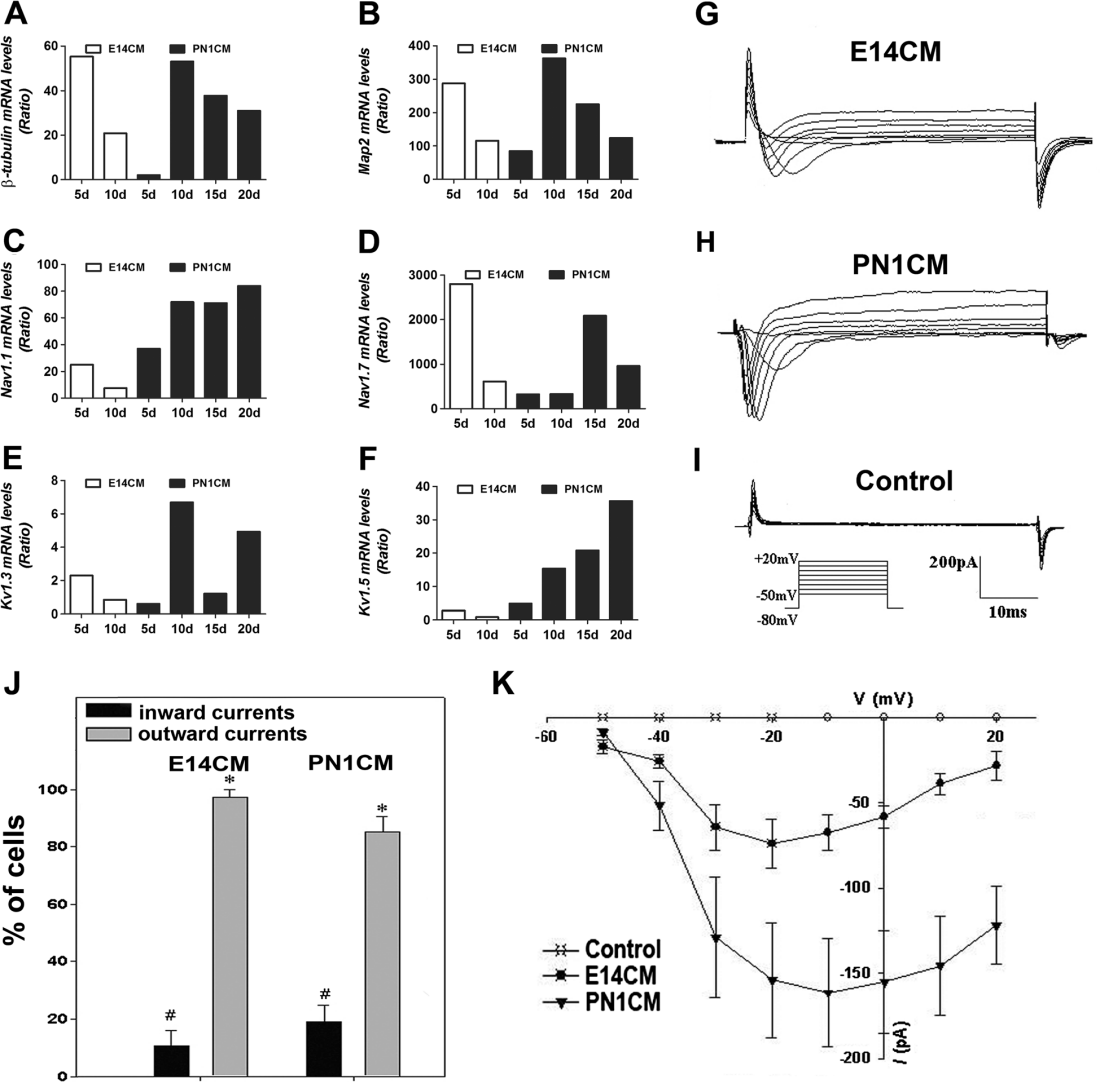
**Cells in CE neurospheres differentiate into functional neurons.** Neurospheres generated by CE stem cells were cultured in the presence of E14CM/PN1CM, and their differentiation into generic neurons was examined. Q-PCR analysis revealed temporal patterns in the acquisition of the expression of neuron-specific marker (*β − tubulin,* p < 0.0001; and *Map2,* p < 0.0001) **(A, B)**, tetrodotoxin-sensitive sodium channel (*NaV1.1,* p = 0.0001*;* and *NaV1.7,* p < 0.0001) **(C, D)**, and potassium channels α subunit (*Kv1.3,* p < 0.0001*;* and *Kv1.5,* p = 0.004) **(E, F)** genes in E14/PN1CM. The levels represent the expression, relative to that in untreated CE cells (ratio). Whole cell voltage clamp recordings revealed fast inward currents in 10.8% (N = 37) of cells in E14CM **(G, J)** and 19.5% (N = 47) of cells in PN1CM **(H, J)**. The current-voltage (I-V) curve **(K)** exhibited a typical I-V relationship of voltage gated Na + channels. Cells in both conditions (>80%; N = 37) displayed sustained outward currents conducted most likely by outwardly rectifying K + channels. These currents were not detected in control CE cells **(I, K)**.

Next, we examined whether or not cells in CE neurospheres could differentiate into generic neurons with functional properties, a lesser burden than becoming highly specialized retinal neurons such as photoreceptors and RGCs. Examination of these cells in differentiation culture conditions consisting of PN1CM/E14CM by Q-PCR, revealed a temporal increase in the levels of transcripts corresponding to *β − tubulin* and *Map2*, markers of generic neurons (Figure 2A and B). The levels fluctuated after the initial increase but remained higher than controls. More importantly, from a functional viewpoint, we observed a similar temporal increase in the levels of transcripts corresponding to *Nav1.1* and *Nav1.7* (Figure 2C and D), encoding a sensitive sodium channel that are broadly expressed in neurons [30,31], and *Kv1.3* and *Kv1.5* (Figure 2E and F), encoding a voltage-sensitive potassium channel which allow neurons to repolarize after action potential, and a delayed rectifying potassium channel, respectively [32,33]. While *Nav1.1* and *Kv1.5* transcripts displayed a steady temporal increase in their levels, those of *Nav1.7* and *Kv1.3* had a less regulated temporal pattern. However, levels of transcripts corresponding to these channels remained significantly higher than controls, except for *Kv1.5* on the 10^th^ day in E14CM. The whole cell patch recording of cells cultured in E14CM that displayed bipolar morphology revealed fast inward currents and sustained outward currents in 10.8% (N = 37) cells (Figure 2G and J). Under similar conditions of recordings, 19.5% (N = 47) of cells cultured in PN1CM displayed fast inward and sustained outward currents (Figure 2H, J and K). The fast inward currents, activated at -40 mV and peaked at -20(E14CM)/-10(PN1CM) mV, exhibited I-V relationship typical of voltage-gated Na + channels (Figure 2K). Currents were not detected in control cells (Figure 2I and K). Together, these observations suggested that a subset of cells in neurospheres, under the influence of specific culture conditions, had differentiated into functional neurons.

**Figure 3:**
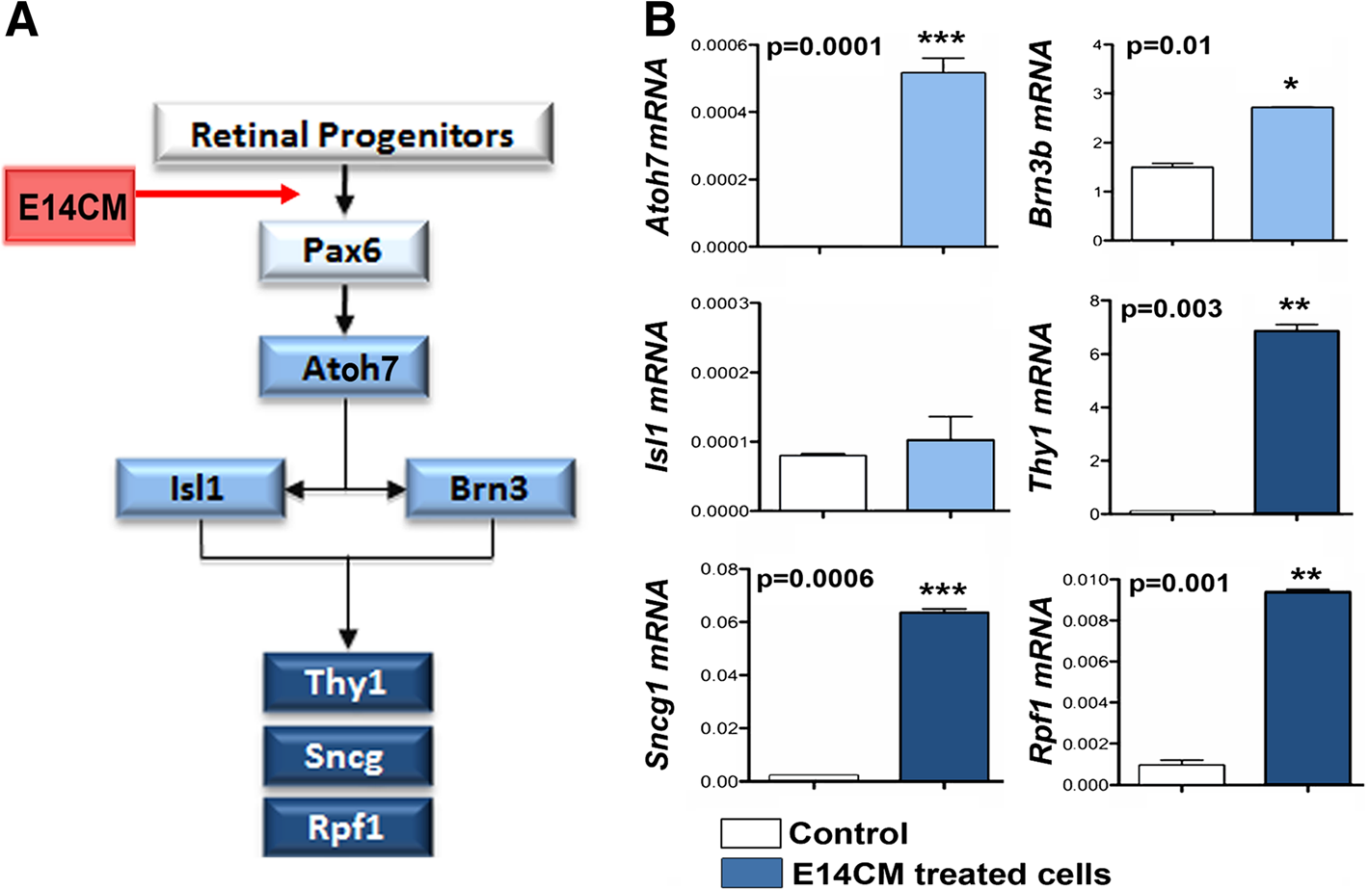
**Cells in CE neurospheres activate RGC-specific regulatory gene expression.** CE neurospheres were cultured in the presence of E14CM for 5 days and RGC regulatory gene expression [37] was examined **(A)**. Q-PCR analysis of cells revealed a significant increase in levels of transcripts corresponding to the regulators of differentiation (*Atoh7* and *Brn3b*) and maturation (*Rpf1, Thy1*, and *Sncg*) of RGCs in differentiation conditions, compared to controls **(B)**. However, no significant induction in the expression of *Isl1* was observed. Controls: CE untreated cells.

**Figure 4:**
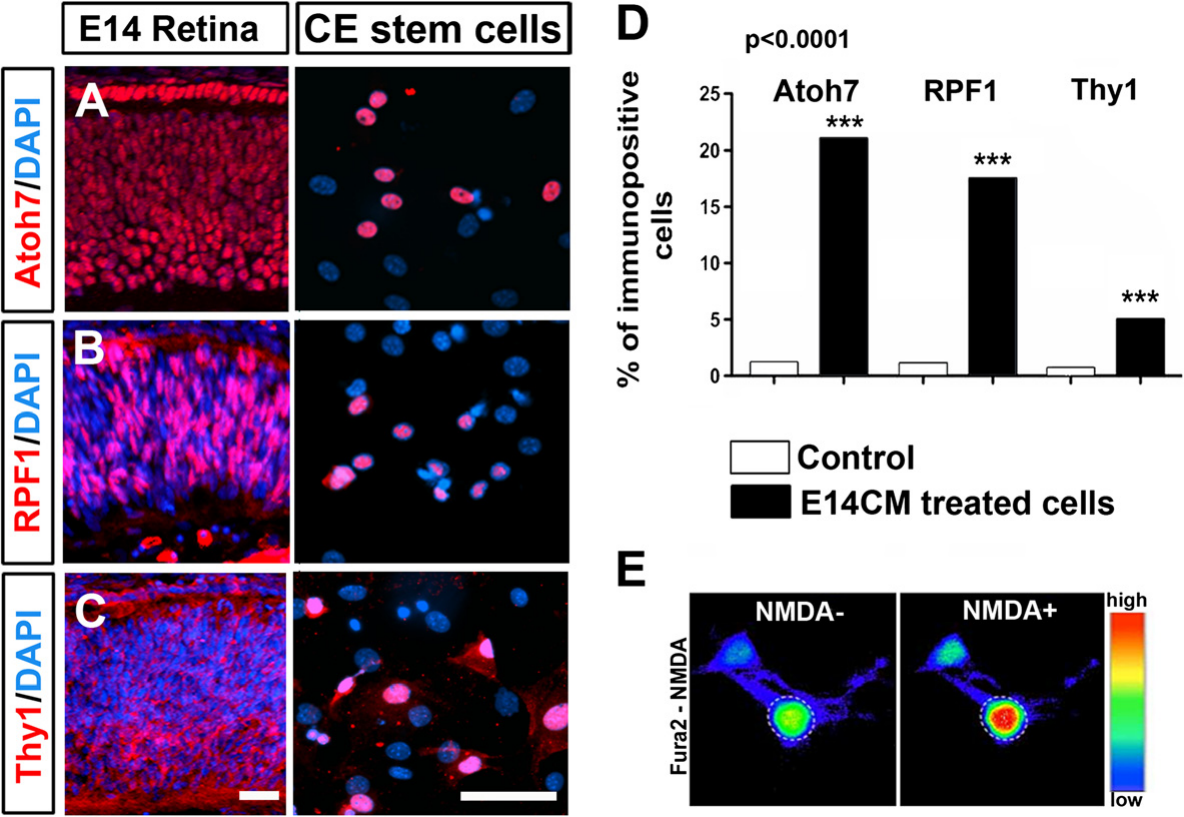
**Cells in CE neurospheres express RGC-specific regulatory and marker proteins.** Immunofluorescence analysis of CE cells in the presence of E14CM for 5 days revealed a subset expressing immunoreactivities corresponding to RGC markers, Atoh7, RPF1 and Thy1, like RGC progenitors/precursors in E14 retina **(A-C)**, the proportion of which was significantly higher, compared to controls **(D)**. Calcium imaging by Fura2 revealed the mobilization of intracellular calcium in differentiated cells in the presence of NMDA agonist (NMDA+) and not in its absence (NMDA-), indicating the presence of ionotropic glutamate NMDA receptor expressed by RGCs *in vivo***(E)**. Bar illustrates fluorescence intensity in a pseudo-color scale **(E)**. Controls: CE untreated cells. Scale = 50 μm.

Next, we determined whether or not cells in CE neurospheres have the capacity to respond to stage specific developmental cues and differentiate along multiple retinal sub lineages, a true measure of their plasticity. First, we examined their potential to differentiate into RGCs, the early born retinal neurons, when neurospheres were cultured in the presence of E14CM, simulating the environment of early retinal histogenesis [34-36] (Figure 3A). Q-PCR analyses of differentiation revealed a significant induction in the levels of transcripts corresponding to genes underlying the regulatory hierarchy of RGC specification and differentiation, *Atoh7* and *Brn3b*[37], compared to controls (Figure 3B). A similar induction of genes, characterizing differentiated RGCs, *Thy1*, *Sncg1*, and *Rpf1*[37] was also observed. However, no significant induction in *Isl1* expression was observed. Immunocytochemical analysis of neurospheres after ten days in culture (Figure 4A-C) revealed a subset of cells expressing immunoreactivities corresponding to Atoh7 (21 ± 1.0%), RPF1 (18 ± 2.5%), and Thy1 (5 ± 1.0%), in proportions that were significantly higher than controls (Figure 4D). These cells, however, did not display the morphology typical of RGCs. Calcium imaging with Fura2 showed rapid increase in intracellular calcium in a subset of differentiated bipolar CE cells with small nuclei when exposed to NMDA, confirming the activity of ionotropic NMDA glutamate receptors, a functional feature of RGCs [38] (Figure 4E). The specificity of differentiation along RGC lineage was further demonstrated by the absence of transcripts corresponding to rod photoreceptors and expression of GFP in differentiated cells (data not shown). Together, these observations suggested that adult CE stem cells have the capacity to respond to developmental cues for early born retinal neurons by activating RGC-specific regulatory genes and differentiate along the RGC lineage.

**Figure 5:**
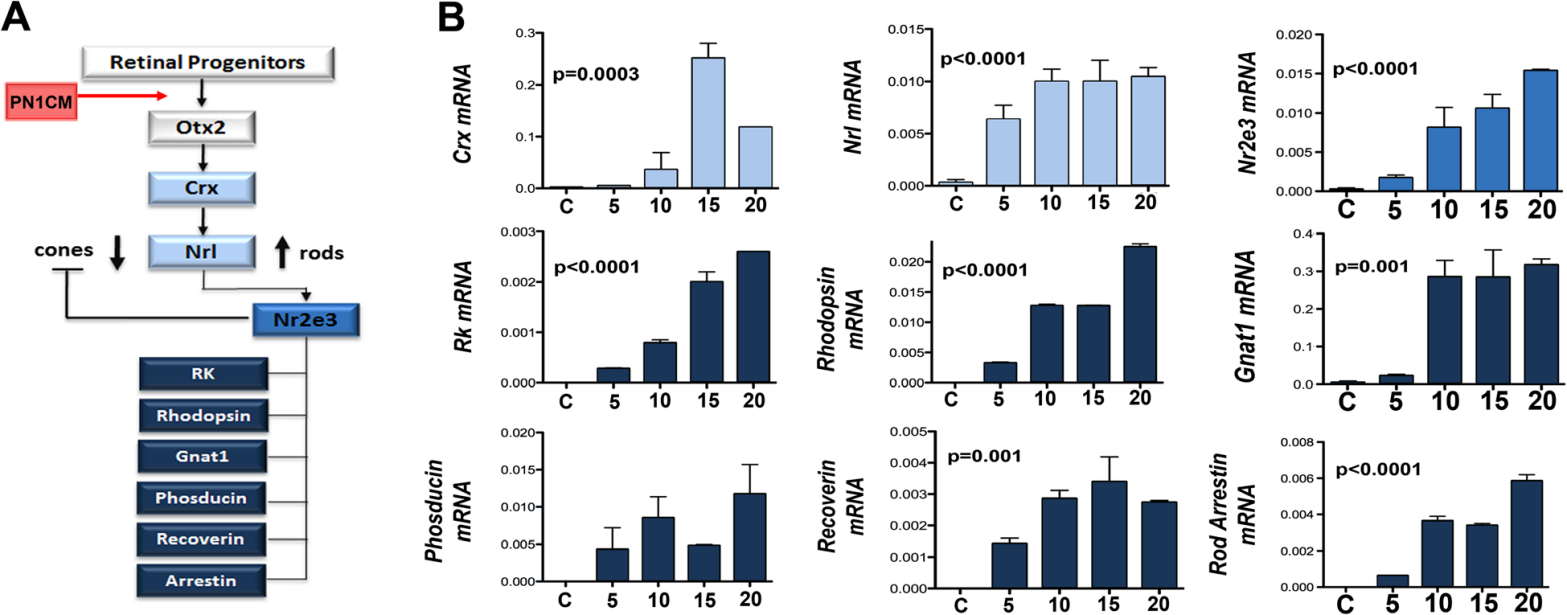
**Cells in CE neurospheres activate rod photoreceptor-specific regulatory gene expression.** CE neurospheres were cultured in the presence of PN1CM for 20 days and rod photoreceptor regulatory gene expression [42] was examined **(A)**. Q-PCR analysis revealed a temporal increase in levels of genes corresponding to the regulators of rod differentiation (*Crx*, *Nrl*, and *Nr2e3*) and maturation (*Rhodopsin kinase*, *Rhodopsin*, *Gnat1*, *Phosducin*, *Recoverin*, and *Arrestin*) in differentiation conditions, compared to controls **(B)**. C = control (CE untreated cells).

**Figure 6:**
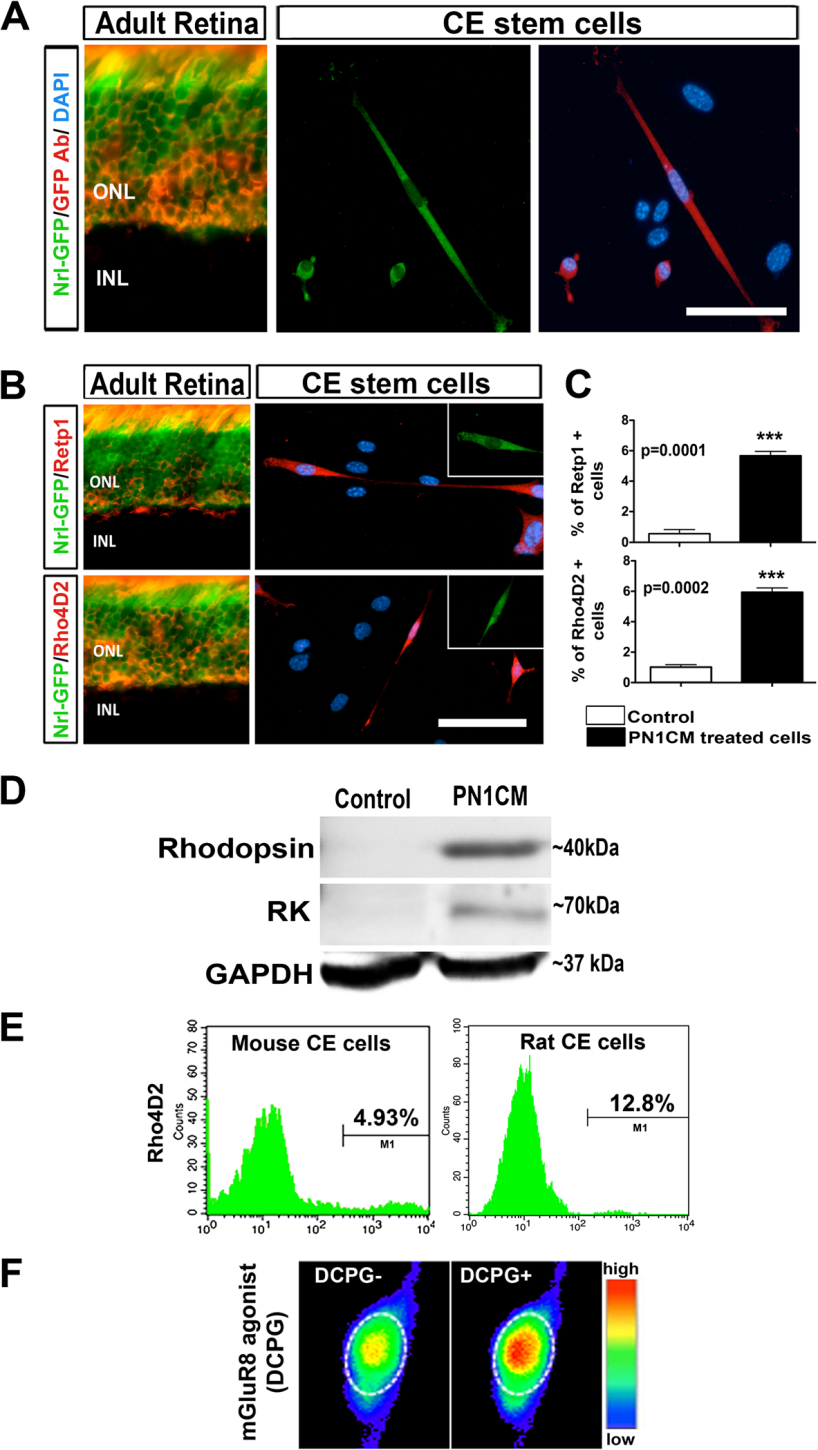
**Cells in CE neurospheres cells express rod photoreceptor-specific regulatory and marker proteins.** After CE neurospheres were cultured in the presence of PN1CM for 20 days, a small subset of cells was positive of GFP fluorescence, indicating the activation of *Nrl* promoter **(A)**. A selected field shows three GFP-positive cells immunoreactive to GFP antibody demonstrating the specificity of the Nrl-GFP-fluorescence, similar to that in the section of PN1 Nrl-GFP mouse retina. That the rare Nrl-GFP-positive cells were of rod photoreceptor lineage was demonstrated by co-localization of rhodopsin immunoreactivities, detected by RetP1 (upper panel) and Rho4D2 (lower panel) with Nrl-GFP fluorescence, as in PN1 Nrl-GFP retinal sections **(B)**. The proportion of cells with rhodopsin immunoreactivities, detected by RetP1 and Rho4D2, was significantly higher in cells in differentiation conditions than in controls **(C)**. Western analysis of cells after 20 days of differentiation revealed 40 kD and 70 kD bands, immunoreactive to Rhodopsin and Rhodopsin Kinase (RK), respectively **(D)**. Examination of species-specific difference in the retinal potential of CE cell revealed mouse and rat CE neurospheres, subjected to identical culture in PN1CM, generate 4.93% and 12.8% of Rho4D2 positive photoreceptors, respectively, on FACS analysis **(E)**. Calcium imaging by Fura2 revealed the mobilization of intracellular calcium by differentiated cells in the presence of agonist DCPG (DCPG+) and not in its absence (DCPG-), demonstrating the presence of mGluR8 metabotropic glutamate receptor, expressed by rod photoreceptors *in vivo***(F)**. Controls: CE untreated cells. Bar illustrates fluorescence intensity in a pseudo-color scale. ONL = outer nuclear layer; INL = inner nuclear layer. Scale = 50 μm.

Next, we examined the potential of cells in adult CE neurospheres to differentiate into late born neurons, rod photoreceptors, when cultured in the presence of PN1CM [39-41], simulating the environment of late retinal histogenesis, when rod photoreceptors are born (Figure 5A). Given the controversy regarding photoreceptor differentiation of CE stem cells [26], we examined gene expression every 5^th^ day for 20 days in culture. We observed a temporal increase in the expression of genes that have emerged underlying the regulatory hierarchy of rod photoreceptors (*Crx, Nrl,* and *Nr2e3*) and those that encode components of photo-transduction machinery (*Rhodopsin*, *Gnat1*, *Phosducin*, *Rhodopsin kinase*, *Recoverin*, and *Arrestin*) [42] (Figure 5B). Examination of differentiation at cellular levels revealed a small subset of cells, majority of them with bipolar cell morphology, expressing GFP, providing a genetic evidence of the activation of *Nrl* promoter (Figure 6A). The expression of GFP was confirmed by immunocytochemical analysis using GFP antibody to rule out the possibility of results due to auto-fluorescence (Figure 6A and Additional file 1). The GFP-positive cells expressed immunoreactivities corresponding to rhodopsin suggesting the maturity of Nrl-positive cells along the rod photoreceptor lineage (Figure 6B). The differentiation along the rod photoreceptor lineage was corroborated and confirmed by the following approaches. First, we carried out immunocytochemical detection of rhodopsin using a different antibody that recognizes a different epitope and observed a similar proportion of cells expressing rhodopsin immunoreactivity (RetP1 = 5.6 ± 0.29%, p < 0.0001; Rho4D2 = 5.9 ± 0.33%, p < 0.0001, Figure 6B and C and Additional file 1). Second, protein (50 ug) obtained from bulk culture of CE neurospheres in PN1CM was subjected to Western analysis (Figure 6D and Additional file 2), which revealed the presence of fractionated proteins immunoreactive to Rhodopsin (40 kD) and Rhodopsin Kinase (70 kD). These immunoreactive bands of proteins were not detected in controls. Third, to determine whether the difference in the efficiency of rod photoreceptor differentiation observed here with mouse CE stem cells compared to that previously observed with rat CE stem cells [4] was not methods/conditions specific, we subjected rat CE cells to identical conditions of differentiation (Figure 6E). We observed a previously reported proportion (~13%) of rat adult CE stem cells expressing rhodopsin [4], suggesting the difference in the efficiency of rod photoreceptor differentiation is species-specific. In these differentiated cells, with altered immunocytochemical conditions, opsin immunoreactivities are detected localized to the membrane (Additional file 1E). Lastly, calcium imaging with Fura2 showed rapid increase in intracellular calcium in a subset of differentiated cells when exposed to DCPG (Figure 6F), indicating the expression of mGluR8 metabotropic glutamate receptors, a functional feature of rod photoreceptors [43,44]. Together, these observations suggested that adult CE stem cells have the capacity to respond to developmental cues for late born retinal neurons by activating rod photoreceptor-specific regulatory genes and differentiate along the rod photoreceptor lineage.

## Discussion

The growth factor-responsive cells in adult CE [45] may represent the evolutionarily conserved counterparts in the ciliary margin zone that sustains regeneration in the lower vertebrates [24,46]. Though the physiological role of these cells remains obscure their presence and accessibility offers an opportunity for their use in regenerative medicine, given their self-renewing capacity and plasticity *in vitro*. Based on their location and stem cell properties that they display *in vitro* they were termed adult CE stem cells [2,22]. Alternatively, based on their progenitor properties and ability to differentiate along retinal lineage *in vitro,* they were characterized as retinal stem cells [1], the existence of which was questioned by Cicero et al., 2009 [26] on the grounds of their pigmentation, epithelial features, and low efficiency of retinal differentiation. Pigmentation was recognized early on as a feature of the epithelium to which adult CE stem cells belonged and their retinal potential was explained based on their reprogramming *in vitro*[2,22]. Such reprogramming, at the light microscopic level, was suggested by the observation that some adult CE stem cells lose their pigmentation as they divided *in vitro*, and that the majority of cells that displayed differentiated phenotypes were apparently devoid of pigmentation [1,2]. In contrast, ultra-structure analyses suggested that the reprogramming might not lead to complete erasure of parental properties as these cells maintained some pigmentation and epithelial features [7,9]. The pigmentation and epithelial features were regarded as contradictory to the nature of stem cells with retinal potential [26]. However, pigmentation as an exclusionary criterion demands caution. For example, metabolic products such as melanin, which are resistant to degradation [47], may not be a reliable indicator of the lack of lineage conversion as they may persist long after the expression of genes associated with their biosynthesis has been attenuated. Also, the presence of stem cell properties and epithelial features such as the presence of adherence junctions, tight junctions, and gap junctions are not mutually exclusive; neuroepithelium and their stem cell derivatives possess these features and their role in the regulation of stem cells, both embryonic and somatic, has begun to emerge [4,48-50]. The presence of morphological features of differentiated cells such as microvilli may not be an exclusionary criterion [26] either, given the evidence that some stem cells, embryonic [51,52] and somatic [53,54] display such features. In the adult retina a subset of highly morphologically and functionally differentiated cells, Müller glia, possess stem cell properties and undergo re-programming to sustain regeneration [46,55].

**Figure 7:**
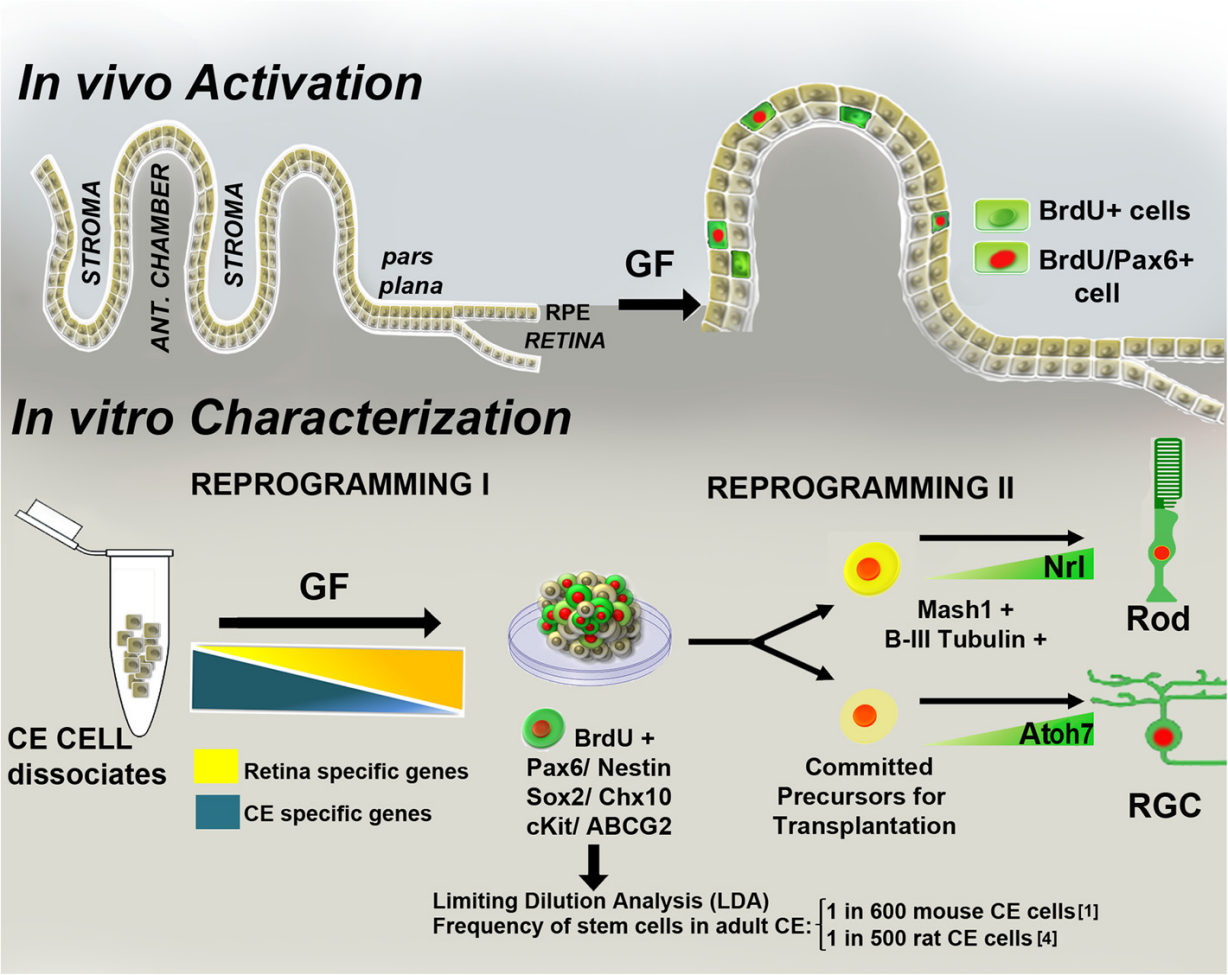
**Schematic representation of putative re-programming of CE stem cells *in vitro*.***In vivo* exposure of adult CE to exogenous growth factors (GF) such as FGF2 and insulin promotes proliferation of quiescent CE stem cells and some of them express Pax6 [45]. Whether or not these cells are capable of generating neurons or more specifically retinal cells is not well known. These cells, when cultured in the presence of mitogens, generate neurospheres accompanied by a decrease in the expression of CE-specific genes and the acquisition of the expression of genes corresponding to retinal progenitors, representing the first stage of reprogramming. The resulting neurospheres consists of heterogeneous population of cells with subsets, which are BrdU positive, and express pan neural and retinal progenitor markers. The frequency of CE cells capable of generating neurospheres is 1 in 600 mouse and 500 rat CE cells, as determined by LDA analyses [1,4]. Cells in neurospheres have the potential to respond to specific cues for retinal differentiation. Therefore, depending upon the cues, they activate expression of regulatory genes for rod photoreceptors (e.g., *Nrl*) or RGCs (e.g., *Atoh7*) to differentiate along specific retinal sub-lineages, representing the second stage of reprogramming. The efficiency and fidelity of retinal lineage conversion will be influenced by the efficiency of reprogramming at both stages. Since the reprogramming is non-cell autonomous, its efficiency will directly depend upon culture conditions, the variability in which might have led to contradictory results.

Our results show that adult CE stem cells undergo two-step reprogramming in response to defined culture conditions (Figure 7). In the first step, adult CE stem cells down regulate the expression of parental genes (*Tyrosinase*, *Palmdelphin*, and *Rab27b*) and progressively acquire of those that encode progenitor regulators (*Otx2, Lhx2,* and *Rx*) in the presence of FGF2 in the culture medium. Expression of *Pax6*, which is detected in adult CE stem cells *in vivo,*[25,56] increases with the time in culture. Reprogramming at this stage unmasks adult CE stem cells’ progenitor properties. Whether or not the reprogramming involves de-differentiation to a primitive stage, characterized by the expression of pluripotency genes, is currently being investigated. In the second stage, they temporally activate hierarchical regulatory gene expression underlying RGC and rod photoreceptor differentiation when exposed to culture medium simulating early and late retinal histogenesis conditions, respectively. Reprogramming at this stage exposed their malleability to non-cell autonomous cues for retinal differentiation. Reprogramming of cells either by alterations of their microenvironment [57] or forced expression of specific transcription factors [58-60] may not extinguish parental gene expression completely, and thus emerges an important question if the residual expression would prevent functional lineage conversion. Our results demonstrate that not only the re-programmed cells are capable of generating functional neurons, as demonstrated by the acquisition of the electrophysiological characteristics, but also retinal neurons with some molecular, biochemical and functional attributes of RGCs and rod photoreceptors. This observation supports a recent finding where adult CE stem cell-derived rod photoreceptor like cells expressed cGMP channels gated by endogenous cGMP, voltage-gated channels for rod maturation, and displayed rudimentary responses to light [10]. However in both cases, despite some evidence of functionality, morphological differentiation, typical of rod photoreceptors, was not observed suggesting that (1) the current differentiation methods are inadequate due to the lack of critical factor(s) and optimal timing or both, required for complete morphological differentiation or (2) signal transduction may occur without morphological differentiation such as the elaboration of outer segments. Functional lineage conversion without complete erasure of parental gene expression is not uncommon. For example, when hepatocyte cells trans-differentiate along the pancreatic lineage, they express insulin yet retaining some expression of hepatocyte-specific genes [61]. Similarly, in the case of retrograde reprogramming of somatic cells toward pluripotency by forced expression of transcription factors, residual expression of parental genes and epigenetic signatures persist and are carried over to differentiated lineages [62,63].

The low efficiency of differentiation along the retinal lineage is not unexpected where reprogramming of heterologous cells are required. If trans-differentiation is invoked, where a post-mitotic adult CE cell converts into a post-mitotic retinal cell without an intermediate step, the built-in stochasticity plus the lack of optimal differentiation conditions will predict a low lineage conversion. If de-differentiation is considered, where a small subset of cells first reverts to a proliferative and developmentally immature stage before differentiating along a particular lineage, their relative enrichment in particular culture conditions will be reflected in the conversion efficiency. In either case, culture conditions will play a critical role in the outcome of the lineage conversion and that may be an explanation for the results of Cicero et al., 2009 [26] and Gualdoni et al. 2010 [27]. It is quite likely that the relatively high efficiency of retinal conversion reported recently by Demontis et al., 2012 [10] is likely to their culture conditions that favored the enrichment of adult CE stem cells. Given the fact that adult CE stem cells are derived from the same embryonic neuroepithelium as retina and their counterparts in lower vertebrates sustain the growth of the retina in adults [24] their differentiation along retinal lineage is not unexpected. Cells from another derivative of ocular neuroepithelium, RPE, possess the capacity to differentiate into retinal neurons *in vitro*, however, this plasticity is limited to embryonic stages [64]. Inter-conversion of cell types within the same lineage is not uncommon. Pancreatic and hepatic cells share the same embryonic lineage and trans-differentiation of hepatic cells into pancreatic cells and vice versa is well documented [65]. Inherent in the use of heterologous stem cells, including cells with pluripotent potential, is the barrier of low efficiency of lineage conversion. The challenge for these cells, including adult CE stem cells, is to increase the efficiency, efficacy, and fidelity of differentiation into specific retinal cell types. If that were achieved, their usefulness for retinal cell therapy would not be in doubt.

## Conclusions

In summary, we have demonstrated that adult mouse adult CE stem cells, isolated by neurosphere culture, undergo reprogramming that attenuates the expression of select adult CE-specific genes and acquire the expression of those that characterize retinal progenitors. A subset of these reprogrammed adult CE stem cells display potential to respond to environmental cues, specific to early and late stages of retinal histogenesis and give rise to RGCs and rod photoreceptors with functional attributes. They also generate functional neurons under non-cell autonomous influence. The wide variation of the efficiency of photoreceptor differentiation of adult CE stem cells or lack thereof is likely due to different culture conditions used in different labs. Indeed, the presence of melanin and epithelial features points toward the property of the epithelium to which these cells belong, but do not exclude their characterization as stem cells, when they display multipotetiality and self-renewal *in vitro*. The potential of adult CE stem cells to differentiate into both early and late born retinal neurons suggests their usefulness for cell therapy for retinal degeneration once the efficiency of non-cell autonomous re-programming is reproducibly increased.

## Methods

### Animals, CE cells and neurospheres assay

This study was ethically approved by the Institutional Animal Care and Use Committee (IACUC), at University of Nebraska Medical Center and Nrl-GFP mice, a gift from Dr. Anand Swaroop [28], were housed and bred in the Department of Comparative Medicine at University of Nebraska Medical Center. Isolation and culture of adult CE cells from adult *Nrl-GFP* mice [28] (6-8 weeks old) was performed as previously described [2,3,19]. Briefly, after eye enucleation, cornea, lens, iris and retina were removed to eliminate any potentially dividing cells from these tissues as contaminants. The pigmented CE was incubated in HBSS, containing collagenase (78 U/ml, Sigma) and hyaluronidase (38 U/ml, Sigma) for 35 min at 37°C, followed by dissociation in 0.25% trypsin, 1 mM EDTA, and 20 mg/ml DNase1 for another 35 min. The presence and absence of *Tyrosinase* (Figure 1) and rod photoreceptor specific transcripts (Figure 3), respectively, by PCR reflected the purity of CE cell dissociates. CE cells were cultured in retinal culture medium (RCM) [4] containing FGF2 (10 ng/ml, R&D Systems) and EGF (20 ng/ml, R&D Systems) for 6 days to generate the CE neurospheres.

### RGC and rod photoreceptor differentiation

For RGC differentiation, primary neurospheres were cultured in CM collected from the culture of embryonic day 14 rat retinas (=E14CM), diluted in RCM (1:1) [4]. For rod photoreceptor differentiation, primary neurospheres were cultured on poly-d-lysine and laminin coated glass coverslip in conditioned medium (CM) collected from the culture of postnatal day 1 rat retina (=PN1CM), diluted in RCM (1:1) [4], supplemented with1% B27 (Invitrogen), DAPT (3 μM; Sigma), Sonic hedgehog (Shh, 3 nM; R&D Systems), Taurine (100 μM; Sigma), all-trans Retinoic acid (500 nM; Sigma), and 2% KOSR (Knockout serum replacement, Gibco). CM was centrifuged and filtered before use to eliminate the possibility of cell contamination.

### Polymerase chain reaction

Total RNA was isolated from the cells using the MiniRNeasy Kit (Qiagen) and cDNA was synthesized as previously described [4]. Specific transcripts were amplified with gene-specific forward and reverse primers (Table 1) by regular PCR using a step cycle program on a RoboCycler (Stratagene) or by Quantitative PCR (Q-PCR) using Quantifast SYBR Green PCR kit (Qiagen) on a RotorGene 6,000 (Corbett Robotics). Q-PCR measurements were performed in triplicate; a reverse-transcription-negative blank of each sample and a no-template blank served as negative controls. Amplification curves and gene expression were normalized to the housekeeping gene *Gapdh*, used as an internal standard.

**Table 1:** List of specific primers

**Gene**	**Sequence**	**Size(bp)**	**T**^**o**^	**Accession N.**
Palmdelphin	F: 5′- ATTCTCTTCCTCTCTCCCTGCTGC -3′	102	55	NM_023245.3
	R: 5′- GCTACCATAAATCAAGGTGCGTCC-3′			
Rab27	F: 5′- AGCCAGCAGAAAAGAAATGTGC-3′	155	55	NM_001082553.1
	R: 5′- ATTGGGGTCAGGGGAGAAAGAC-3′			
Tyrosinase	F:5′-CTGTGCCTCCTCTAAGAACTTGTTG-3′	163	57	NM_011661.4
	R:5′-ACGGTCATCCACCCCTTTGAAG-3′			
Ki67	F:5′CCAGAGCTAACTTGCGCTGAC-3′	148	54	NM_001081117.2
	R:5′GCTTGATGGTGACCAGGTGAG-3′			
Cyclin D1	F:5′-ACCCTGACACCAATCTCCTCAAC-3′	118	56	NM_171992
	R:5′-ATGGATGGCACAATCTCCCTCTGC-3′			
Otx2	F:5′-TCTGGTCTCACTCCATCCCC-3′	172	51	NM_144841.3
	R:5′-TGGTTTACTGCTTCGGAGGG-3′			
Lhx2	F:5′-GAGACGTGCAGGCATCTGG-3′	133	55	NM_010710.3
	R:5′-CATCGCTAGCTGGGTTCTGG-3′			
Pax6	F:5′-CACCAGACTCACCTGACACC-3′	193	54	NM_001244198.1
	R:5′-TCACTCCGCTGTGACTGTTC-3′			
Rx	F:5′-ATCCCAAGGAGCAAGGAGAG-3′	256	58	AF135839
	R:5′-TTCTGGAACCACACCTGGAC-3′			
β-III Tubulin	F:5′-TTTTCGTCTCTAGCCGCGTG-3′	157	54	NM_023279.2
	R:5′-CTGCAGGTCTGAGTCCCCTA-3′			
Map2	F:5′-ATTAACCAACCACTGCCGGA-3′	188	52	NM_001039934.1
	R:5′-ATTTGTACATTTCCGCCCCC-3′			
Nav 1.1	F:5′- CATACATCTTTCGGGGGAATCTC-3′	195	54	NM_018733.2
	R:5′- CATCTTTTTTGTCTGGCTTGGG-3′			
Nav 1.7	F:5′- GAAGACCCCGAAGAAGAAGAAGG-3′	194	54	NM_009135.2
	R:5′-GCATTGAACCTGAAGATGACTCTGC-3′			
Kv 1.3	F: 5′- CGAGCGTGTGGTCATCAACATC-3′	128	58	NM_008418.2
	R: 5′- CATTGCGGAGTGGGTCAAAG-3′			
Kv 1.5	F: 5′- CATCAAGGAAGAGGAGAAGCCC -3′	127	56	NM_145983.2
	R: 5′- GAGAATGACCAAGACCGACACG -3′			
Ath5	F:5′- CAGGACAAGAAGCTGTCCAA-3′	173	56	AF071223
	R:5′- GGGTCTACCTGGAGCCTAGC-3′			
Brn3b	F:5′-AGACTTCGAGCAGGAGATG-3′	322	60	NM_138944.2
	R:5′-CTTGATCTTCATGGTGCTAGG-3′			
Isl1	F:5′- TTCTCCGGATTTGGAGTGGC-3′	188	53	NM_021459.4
	R:5′- CACGCATCACGAAGTCGTTC -3′			
Thy1	F:5′- ACCAAGCCAGATGCCTGAAA -3′	147	54	NM_009382.3
	R:5′- GGATGGTCCACAAAGGCTCA-3′			
Sncg1	F:5′-AAGAGGCAGTAGCAGCAGAGACAG-3′	131	56	NM_011430.3
	R: 5′- CAACACCTTCCTTGGCAATGG-3′			
Rpf1	F:5′- AGTTACTAATGCACAAGGACA-3′	380	57	NM_010127.3
	R:5′-CTCAAAGCTGAAGAAGAGGAG-3′			
Crx	F:5′-CCTGTAAAAGAACTGACAAGAGGGG-3′	153	55	NM_001113330.1
	R:5′- AAGGCATTGACTGAATAGTGAGGC-3′			
Nrl	F:5′-TTCTGTCTCATCTTCCAGGCTGTAG-3′	120	50	NM_001136074
	R:5′-ACAACTTCTTCTCCCCCTTCCTC-3′			
Nr2e3	F:5′-CCGAAACTTGTGCTAAACTGGAGC-3′	183	56	NM_013708.4
	R: 5′- GGAAAGGCAGGTTGGAAAACAC-3′			
RK	F:5′-GCTGAACAAGAAGCGGCTGAAG-3′	238	56	NM_011881
	R:5′-TGCTGTGTAGTAGATGGCTCGTGG-3′			
Rhodopsin	F:5′-TCAAGCCTGAGGTCAACAAGC-3′	422	62	BC013125
	R:5′-ACTTCCTTCTCTGCCTTCTGAGTG-3′			
Gnat1	F:5′- AGATGAAGATTATCCACCAGGACG-3′	136	55	NM_008140.2
	R:5′- TCTCCATACTGAATGTTGAGCGTG-3′			
Phosducin	F:5′-TGCTGTGGATGTGGAGTCTTTCC-3′	105	51	NM_001159730.1
	R:5′-GCTTATTCAATGTCCTCGTCTTCC-3′			
Recoverin	F:5′-GGAAAAGAAACAGTGATGGGCAC-3′	188	57	NM_009038.2
	R:5′-AAAAGGAAGCAGGAGTTGTGTCC-3′			
Rod Arrestin	F:5′-TGTCCTCACCCAACTCCAAGAGAG-3′	153	56	NM_009118.2
	R:5′-ACCTCAAAGTCAACCCCACAGC-3′			
GAPDH	F: 5′-ACAGTCCATGCCATCACTGCC -3′	266	60	NM_017008.3
	R: 5′-GCCTGCTTCACCACCTTCTTG-3′			

### Immunofluorescence analysis

Immunofluorescence analysis was performed as previously described [4]. Briefly, paraformaldehyde-fixed cells were incubated in 1xPBS containing 5% NGS and 0.2 or 0.4% Triton X-100, followed by an overnight incubation with the antibodies (Table 2). Cells were examined for epifluorescence after incubation with anti–species-specific immunoglobulin G conjugated to Cy3 or FITC. Images were captured using a cooled CCD camera (Princeton Instruments) and Openlab software (Improvision) using Leica DMR or Zeiss 510 Meta Confocal Laser Scanning Microscope.

**Table 2:** List of primary antibodies

**Name**	**Species**	**Dilution**	**Company**
Ret-P1	Mouse	1:100	Gift
Rho4D2	Mouse	1:100	Gift
Rhodopsin Kinase	Mouse	1:200	Affinity Bioreagents (554895)
Rx	Rabbit	1:100	Santa Cruz (sc-79031)
Pax6	Rabbit	1:100	Covance (PRB-278P)
Atoh7	Rabbit	1:100	Millipore (AB5694)
RPF1	Mouse	1:200	Gift
Thy1	Rabbit	1:100	BD Pharmingen (554895)
GFP	Rabbit	1:200	Millipore (AB3080P)

### Western analysis

The protein samples (50 μg), extracted from cultured cells using T-PER Tissue Protein Extraction Reagent (Thermo Scientific), were denatured and separated by sodium dodecyl sulfate-polyacrylamide gel (12%) electrophoresis, and then transferred onto the 0.45 micron PVDF-Plus Transfer Membrane (GE Water & Process Technologies). Membranes were blocked in TBS-Tween (25 mm Tris-HCL, pH 8.2, 144 mm NaCl, 0.1% Tween-20), with 5% skim milk and 1% BSA for 2 hours at room temperature, followed by incubation with primary antibodies (Table 2) overnight, at 4°C. Membranes were washed and incubated with HRP-conjugated secondary antibodies for 1 hour at room temperature, and visualized with an enhanced chemiluminescence reagent (ECL Plus Western Blotting Detection System, Amersham).

### FACS analysis

Cells were dissociated by trypsinization (0.25%) for 10 min, washed with 1X PBS, and fixed with 4% paraformaldehyde for 15 minutes, followed by permeablization in 0.2% saponin, containing 5% NGS, for 30 min. Cells were incubated, first with respective primary antibodies (Table 2) for 2 hours and washed with PBS containing 0.2% saponin and then with secondary antibody conjugated to Cy5 for 1 hour, followed by analysis on an LSRII Flow Cytometry System (BD Biosciences).

### Calcium imaging

Differentiated cells were incubated in culture medium containing acetoxy-methyl ester Fura-2 (5 μM, Invitrogen) for 30 min at 37°C. After a 15-minute rinse in culture medium without Fura-2 AM the coverslip was mounted on the perfusion chamber, fitted on an inverted Olympus microscope (IX70). Test solutions [20 mM (S)-3,4-dicarboxyphenylglycine (DCPG); 100 mM (RS)-alpha-cyclopropyl-4-phosphonophenylglycine (CPPG), and 200 nM N-methyl-D-aspartate (NMDA), Tocris,) were applied by bath perfusion. Cells were subjected to excitation wavelength at 340 and 380 nm, using Lamda DG-4 (Sutter Instrument). Fluorescence changes were monitored every 5 seconds by cooled charge-coupled device (CCD) camera (Orka II, Hamamatsu) and ratio-metric imaging carried out using Open Lab Software (Improvision).

### Electrophysiological analysis

Membrane currents were recorded under whole-cell patch configuration [4]. The cells were voltage clamped at the steady membrane potential of -80 mV and currents were induced by voltage steps (-80 mV to -50 mV in the first step, then to +20 mV in increments of 10 mV). The bath (extracellular) solution contained (mM): NaCl, 160; KCl, 4.5; CaCl_2_, 2; MgCl_2_, 1; HEPES, 5; glucose, 11; adjusted to pH 7.3 with NaOH. The pipette solution contained (mM): KCl, 150; CaCl_2_, 1; MgCl_2_, 2; EDTA, 11; HEPES, 10; adjusted to pH 7.3 with KOH. Experiments were performed at room temperature. The patch pitettes of 2-5 MΩ were fabricated on a two-stage puller (PC-10, Narishige). Currents were amplified using an Axopatch 200B amplifier (Axon instruments), filtered at 1 kHz, digitized at 5 kHz using a digital 1440A digitizer, and recorded using pClamp10 software. Junction potentials were corrected and the cell capacitance was compensated (~70%) in all cells tested.

### Statistical analysis

Cell type-specific antigen-positive cells were counted in 10-15 randomly selected fields in three to five different coverslips. Each experiment was repeated at least three times. Values were expressed as ± SEM. Data were analyzed using the Student’s t-test or ANOVA to determine the significance of the differences between treatment and control groups.

## Abbreviations

CE: Ciliary epithelium; RGCs: Retinal ganglion cells; RPE: Retinal pigmented epithelium; RCM: Retinal culture medium; CM: Conditioned medium; PN1CM: Postnatal day 1 conditioned medium; E14CM: Embryonic day 14 conditioned medium; Q-PCR: Quantitative PCR; kD: kilodalton; DCPG: (S)-3,4-dicarboxyphenylglycine; NMDA: N-methyl-D-aspartate; GFP: Green fluorescence protein.

## Competing interests

The authors declare no conflict of interest.

## Authors’ contributions

DD, CB: Collection and assembly of data, data analysis and interpretation, manuscript writing. XP: Collection and assembly of data. XH: Data analysis and interpretation. AI: Conception and design, data analysis and interpretation, manuscript writing. All authors read and approved the final manuscript.

## Supplementary Material

Additional file 1 **Protein profile of CE photoreceptors’ differentiation.** CE neurospheres were cultured in the presence of PN1CM for 20 days. Immunostaining indicated a small subset of cells with GFP fluorescence (Nrl-GFP, green) demonstrating the activation of Nrl promoter, immunoreactive to GFP antibody (GFP-Ab, red), indicating the specificity of the Nrl-GFP-fluorescence (A,B). Co-localization of Nrl-GFP fluorescence (green) and rhodopsin immunoreactivities (red), detected by RetP1 (C) and Rho4D2 (D). Detailed immunostaining on Retp1 positive cells indicating membrane localization of rhodopsin (E) in differentiated rat CE cells. Click here for file

Additional file 2 **Western blot analysis of differentiation of CE stem cells along the rod photoreceptor lineage.** CE neurospheres were cultured in the presence of PN1CM for 20 days. Western blot analysis revealed the presence of ~40 kD and ~70 kD immunoreactive bands, corresponding to Rhodopsin (A) and Rhodopsin Kinase (RK) (B), respectively, among other bands in protein samples extracted from the adult retina, untreated CE cells, and CE cells cultured in the presence of PN1CM. Click here for file

## Acknowldgements

Thanks are due to Dr. Sowmya Parameswaran, Ramkishore Gernapudi, Ani Das, Chandrika Abouri and Graham Sharp for help in the CE stem cells project and critical reading of the manuscript. This work was supported by Lincy Foundation, Pearson Foundation, and Otis Glebe Foundation.
