# Supplementary figures and images for: Adult ciliary epithelial stem cells generate functional neurons and differentiate into both early and late born retinal neurons under non-cell autonomous influences
Source: BMC Neurosci. 2013 Oct 22;14:130. doi: 10.1186/1471-2202-14-130 (PMC3856605; doi:10.1186/1471-2202-14-130)

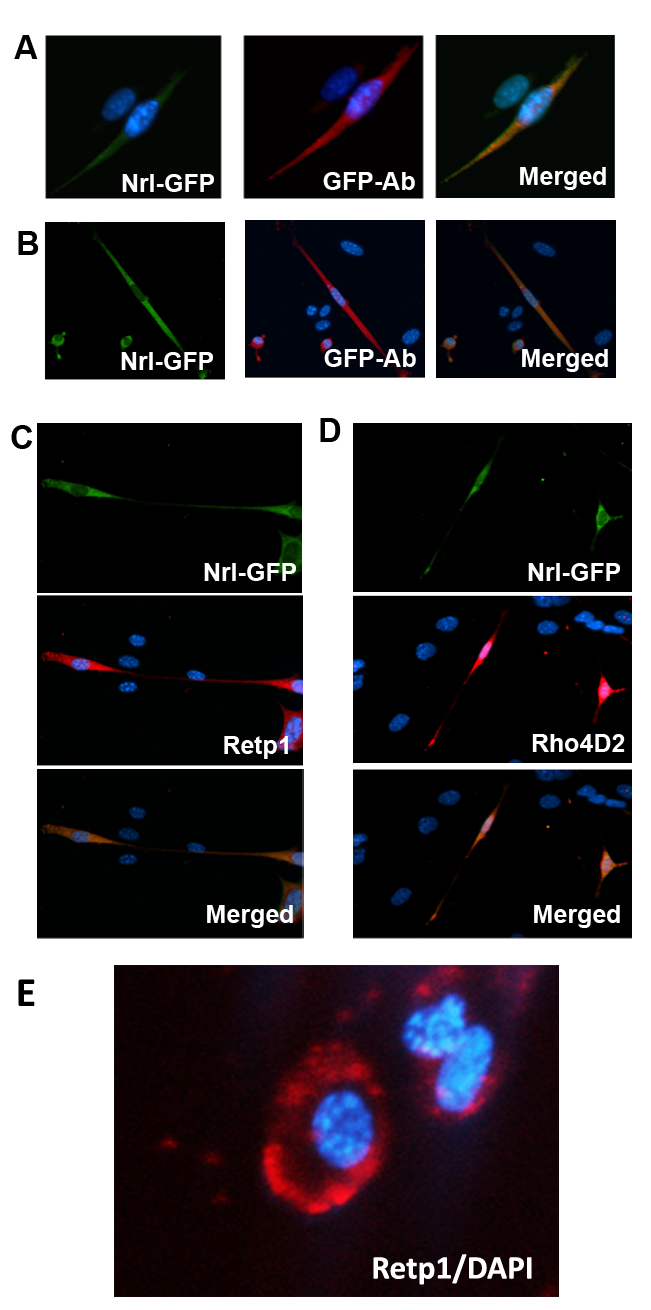

Supplement: Additional file 1 — Protein profile of CE photoreceptors’ differentiation. CE neurospheres were cultured in the presence of PN1CM for 20 days. Immunostaining indicated a small subset of cells with GFP fluorescence (Nrl-GFP, green) demonstrating the activation of Nrl promoter, immunoreactive to GFP antibody (GFP-Ab, red), indicating the specificity of the Nrl-GFP-fluorescence (A,B). Co-localization of Nrl-GFP fluorescence (green) and rhodopsin immunoreactivities (red), detected by RetP1 (C) and Rho4D2 (D). Detailed immunostaining on Retp1 positive cells indicating membrane localization of rhodopsin (E) in differentiated rat CE cells. [file 1471-2202-14-130-S1.tiff]

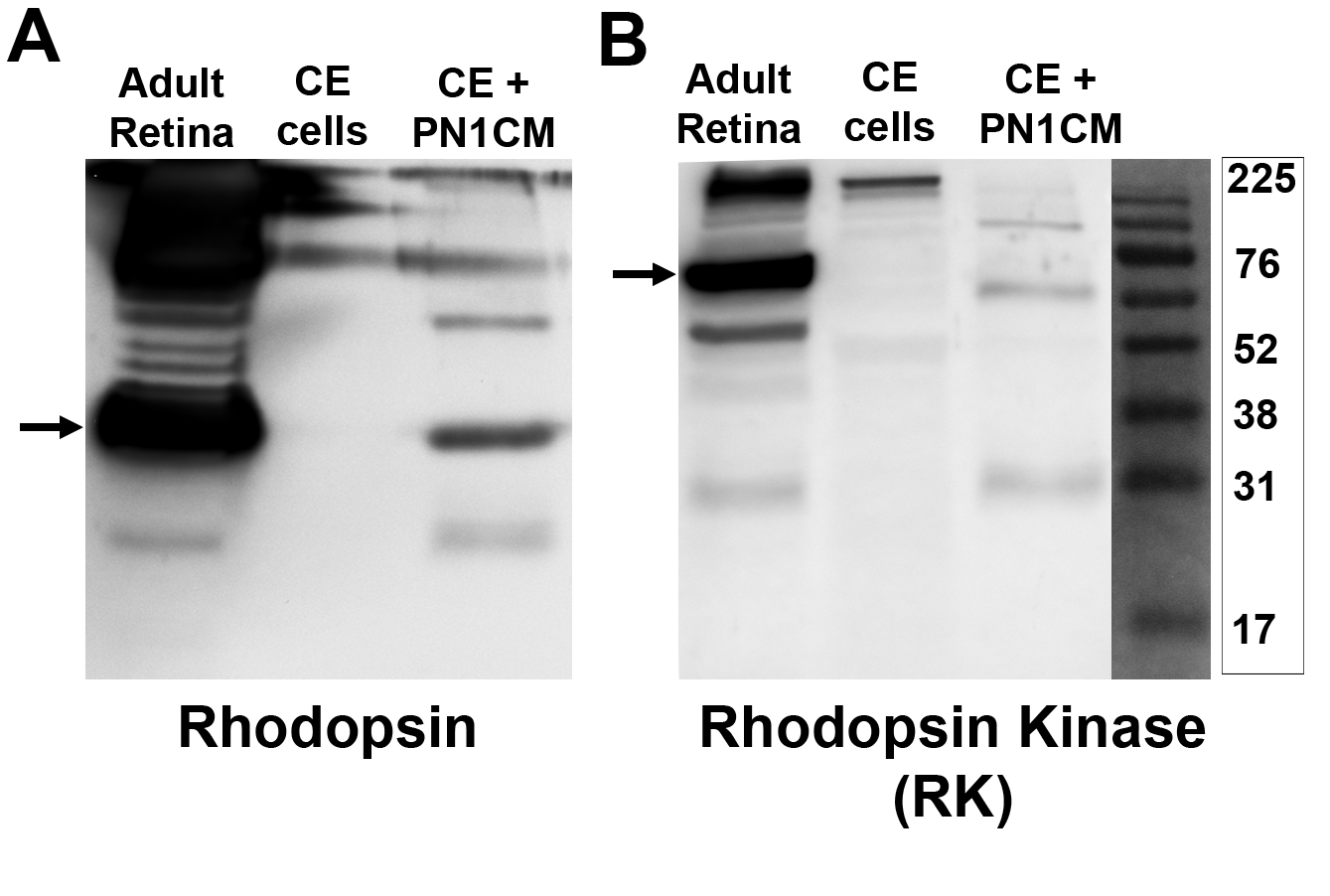

Supplement: Additional file 2 — Western blot analysis of differentiation of CE stem cells along the rod photoreceptor lineage. CE neurospheres were cultured in the presence of PN1CM for 20 days. Western blot analysis revealed the presence of ~40 kD and ~70 kD immunoreactive bands, corresponding to Rhodopsin (A) and Rhodopsin Kinase (RK) (B), respectively, among other bands in protein samples extracted from the adult retina, untreated CE cells, and CE cells cultured in the presence of PN1CM. [file 1471-2202-14-130-S2.tiff]
